# Toward a systematic conflict resolution framework for ontologies

**DOI:** 10.1186/s13326-021-00246-0

**Published:** 2021-08-09

**Authors:** C. Maria Keet, Rolf Grütter

**Affiliations:** 1 Department of Computer Science, University of Cape Town, 18 University Avenue, Cape Town, 7700 South Africa; 2 Swiss Federal Research Institute WSL, Zürcherstrasse 111, Birmensdorf, CH-8903 Switzerland

**Keywords:** Ontology engineering, Ontology development, Infectious disease ontologies

## Abstract

**Background:**

The ontology authoring step in ontology development involves having to make choices about what subject domain knowledge to include. This may concern sorting out ontological differences and making choices between conflicting axioms due to limitations in the logic or the subject domain semantics. Examples are dealing with different foundational ontologies in ontology alignment and OWL 2 DL’s transitive object property versus a qualified cardinality constraint. Such conflicts have to be resolved somehow. However, only isolated and fragmented guidance for doing so is available, which therefore results in *ad hoc* decision-making that may not be the best choice or forgotten about later.

**Results:**

This work aims to address this by taking steps towards a framework to deal with the various types of modeling conflicts through meaning negotiation and conflict resolution in a systematic way. It proposes an initial library of common conflicts, a conflict set, typical steps toward resolution, and the software availability and requirements needed for it. The approach was evaluated with an actual case of domain knowledge usage in the context of epizootic disease outbreak, being avian influenza, and running examples with COVID-19 ontologies.

**Conclusions:**

The evaluation demonstrated the potential and feasibility of a conflict resolution framework for ontologies.

## Background

Use of ontologies keeps gaining traction, which motivates more ontology development and therewith the prospects and task of reusing existing ontologies in whole or in part. Reuse is not only less demanding on the resources than starting with a clean slate and re-inventing the wheel, but it also may foster interoperability across information systems. It is already a key component of the OBO Foundry approach for bio-ontologies [1] and it is incorporated in ontology development methodologies such as NeON [2]. The concrete steps for reuse could involve redesign of a single ontology or the importing or merging of one ontology with another ontology or it may be added to a complex network of integrated ontologies. Some of the myriad examples of different strategies are the top-down approach with the Infectious Diseases Ontology (IDO) within the OBO Foundry [3] and the re-configurable BioTop where modules can be exchanged thanks to multiple alignments [4]. Assessing the potential for (re)use can be difficult, where even one choice can lead to further research, such as which parthood [5, 6], or avail of software-based assistance to choose quickly [7].

A candidate ontology for reuse may not have all the desired axioms or have much more than needed, and, once imported and aligned, may result in an inconsistent or incoherent ontology or be beyond the desired OWL species, or otherwise incompatible. Reuse experiences vary widely also in the biology domain; recent examples include reuse of the IDO with a simulation modeling ontology together with schistosomiasis knowledge [8], the modular design and many reuses of the Gene Ontology [9], and examining subtle differences across disease ontologies even before reuse [10]. For instance, two domain ontologies each may be aligned to a different foundational ontology, which may have representational differences where one ontology has a property vaccinates but the other uses a class Vaccination where they intended to mean the same general notion but one chose the process and the other its reified variant, or there are subject domain disagreements, like one having asserted that Virus is an organisms and the other does not.

Although it can seem overwhelming to assess extant ontologies and to just discard them to start from scratch again, we assume that a modeler would not wish to duplicate work and rather attempt to resolve any issues that may arise. How should one do this? Currently, this proceeds on an *ad hoc* basis, where one may not even be aware of what one should be looking for until the problem manifests itself. There are a few tools that assist with detecting conflicts, such as the explanations generated in Protégé [11], checking the differences in inferences obtained [12], the OWL Species Classifier^1^ that pinpoints which axiom(s) violate which OWL species, and one can test if adding a particular axiom is going to cause problems before actually adding it [13]. Such tools, however, do not detect all sources of conflicts, such as between fundamental assumptions about a domain or preferred theories; e.g., the choice between either parthood or connection or both as primitive for a mereotopological theory [14] and whether it is “better” for one’s domain to declare parthood transitive or use it in qualified number restrictions since one cannot have both in OWL 2 DL [15]. It then requires an overview of the options and consequences, as illustrated next.

### **Example 1**

An OWL ontology *O*_1_ about anatomy has declared that a *biped* is an animal that *has*_*part* exactly two legs ($\mathsf {biped \sqsubseteq animal \, \sqcap =2 \, has\_ part.leg}$). When this o1:*has*_*part* is aligned to the DOLCE ontology in OWL, a tool such as Protégé will report a clash, due to that *dolce*:*has* - *part* is declared as transitive, and therefore the default installed automated reasoner will not work. What can the modeler do? Their main options are:

- do not align to DOLCE;
- give up on the qualified cardinality constraint and modify the definition of *biped*;
- import DOLCE and remove transitivity, rendering it *de facto* incompatible with DOLCE;
- accept to go beyond OWL 2 DL and use a different logic with little to no tool support; or,
- forsake automated reasoning over one’s ontology.

The options have consequences that are all less optimal compared to the (impossible) intention.

The consequences of each choice still have to be assessed in some way, which may leave the ontologist to resort to trial and error attempts, which hampers redeployment of ontologies, also because the consequences of possible solutions may not be clear.

We aim to ameliorate these issues by devising an approach for *meaning negotiation* and *conflict resolution* in the ontology development and (re)use processes. The possible principal sources of conflict for both individual ontology development and networked multiple ontologies are examined. For each underlying source and type of conflict, there is a fixed set of feasible solution strategies, which will enable automatic generation of explanatory implications. Some of the components of the conflict resolution process can be computed automatically, but it is unavoidable to keep a human-in-the-loop who makes the final, but now well-informed, decision. The approach is illustrated and evaluated with a case study of ontology reuse to manage an epizootic disease outbreak (avian influenza) in Switzerland, involving negotiation and resolving conflicts concerning the suitable mereotopological theory and trade-offs with OWL species. Smaller examples throughout the paper are drawn from COVID-19 ontologies.

This paper extends the authors’ ICBO2020 paper [16] in a number of ways. It contains a larger preliminary library of conflicts, an extended case study, more examples with existing ontologies, and a conflict resolution walk-through with a corresponding specification of software requirements.

The remainder of the paper is structured as follows. In the “Methods” section, an approach to meaning negotiation and conflict resolution is introduced. The use case is presented in the “Results” section. The “Discussion” section zooms in on system requirements for software support for conflict negotiation. “Conclusions” are drawn in the last section.

## Methods

We first distinguish between meaning negotiation and conflict resolution. Subsequently, we introduce a preliminary ‘library’ of conflicts and the *conflict set* data structure that stores the minimum necessary data about such conflicts to be used for resolution, and then proceed to resolution strategies.

### Characterizing meaning negotiation and conflict resolution

Negotiating the meaning of the knowledge—classes, properties, and axioms—to be represented in an ontology involves reaching an agreement on: 1) the exact elements required, 2) the domain theory that will provide these elements, and 3) the required ontology language to represent the former. Each item may involve *meaning negotiation* and *conflict resolution*. This will be disambiguated and illustrated first, after which potential sources of conflict are identified and the conflict set is introduced.

#### Types and sources of conflicts

We define informally the concepts of meaning negotiation and conflict resolution, which will be illustrated afterward. **Meaning negotiation** concerns deliberations to figure out the precise semantics one wants to represent in the ontology. They are all *positive choices* in the sense of “which of the options is applicable? Then we take that one”. **Conflict resolution** concerns choosing one option among a set of two or more options, where that choice is deemed the *‘lesser among evils’ for that scenario*. It necessarily involves a compromise and making it work requires reengineering something in at least one of the ontologies or as a whole. Subtypes include: **Language conflict resolution** A conflict arises within the same family of languages or with a m ore distant one. This is either a zero-sum game (i.e., with a winner and a loser) or there may be a joint outside option. **Ontological conflict resolution** The ontologies adhere to different theories, which may be foundational philosophical decisions that affect the overall structure of the ontology or subject domain arguments with competing theories. This is likely a zero-sum game of either-or (no joint outside option).

They are illustrated in the following example.

##### **Example 2**

Meaning negotiation may involve assistance with explanations for the modeler, such as when they do not know whether to give up reflexivity for scalability, to offer them a dialogue alike “*if you don’t have* reflexivity, *you’ll miss these sort of inferences:* [example goes here]”, or one can frame negotiation of alternatives as an imperative, e.g., “*take either* parthood *or* proper parthood as primitive *for your* mereological theory, *but not both.*”.

Conflict resolution applies in many cases; e.g., there are several common types of language conflicts, such as:

- A conflict within a language family, such as the Description Logics-based OWL species, is transitivity versus qualified cardinality constraints, because one cannot have both declared on the same object property, as illustrated in the Introduction with *biped* and *has* - *part*.
- A syntax-level conflict, which manifests itself when having to merge an ontology represented in the common logic interchange format (CLIF) and another one represented in OWL, or OBO and OWL.
- A conflict about a language’s semantics (on top of the syntax issues): when ontologies are represented in different languages where one has a model-theoretic semantics and the other a graph-based one, or even the same overall semantics but one has the unique name assumption (UNA) embedded in the language and the other does not.

What to do then? Besides choosing either, there may be a so-called ‘joint outside option’ (a term from game theory) where neither wins, but there is an alternative option. For instance, instead of debating over transitivity vs qualified cardinality, leave OWL to choose CLIF, or when deliberating CLIF or OWL in an either-or way, one can keep both and move outside either setting and into the framework of the Distributed Ontology, Model and Specification Language (DOL) [17], which is a meta-language that provides the ‘glue’ between ontologies represented in different languages, including more expressive ones. This strategy was illustrated in [5] for mereotopological theories.

The language’s semantics can be an example of a zero-sum game, such as to either embed the UNA in the semantics or not, but it cannot be both, and it affects the reasoner’s deductions. For instance, consider some ontology O1 that contains $\mathsf {C \sqsubseteq \,\, =1\, R.D}$ together with the assertions {R(a1,b1),R(a1,b2),C(a1),D(b1),D(b2)}. Under no-UNA such as with OWL, it will deduce b1=b2, because it is the only way to satisfy the =1 constraint; under UNA, it will deduce the ontology is inconsistent because is violating the =1 constraint in the TBox.

An example of an ontological conflict is a clash in the top-level organization of the ontology, such as between BFO and GFO, and related philosophical differences, such as qualities with qualia vs trope theory to represent attributes [18]. At the subject domain level, this may be, e.g., whether a virus is a living thing or not and competing scientific theories more generally. They do not have a joint outside option because the theories conflict, except returning it to the domain experts to resolve (e.g., to conduct experiments).

**Fig. 1:**
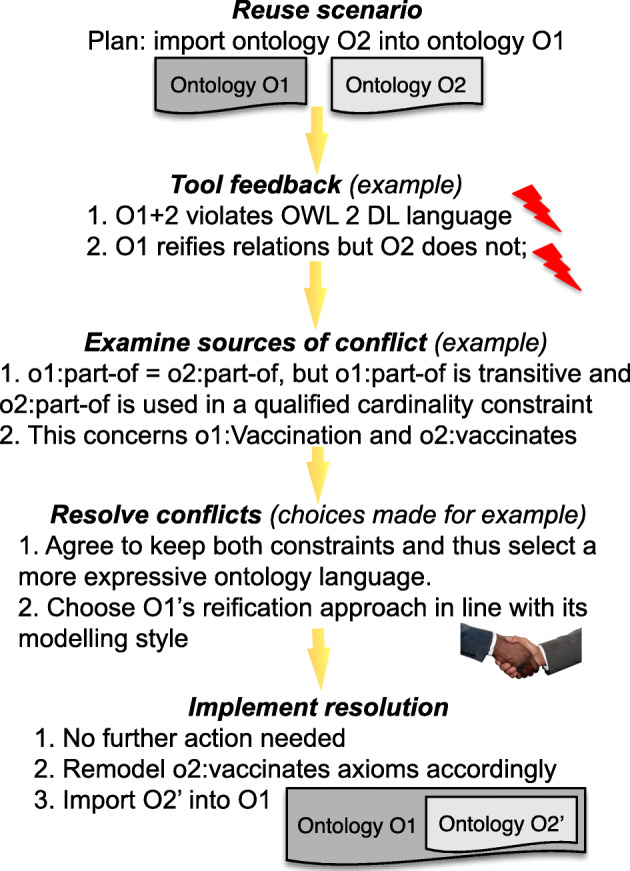
Sample scenario (summarized) to detect and resolve conflicts in an ontology reuse scenario where the intention is to import ontology O2 into ontology O1
- *Logic limitations causing conflicts for an ontology, affecting the software ecosystem*; e.g., the biped’s *has* - *part* being either transitive or have it participate in axioms with qualified cardinality constraints in OWL 2 DL, or facing this clash when trying to merge or integrate two ontologies.
- *Logic limitations by design, for the purpose of scalability*; e.g., there are axioms in one’s ontology that are beyond the desired OWL species, so that one has to choose either to abandon the preferred species or to remove the axioms.
- *Certain deductions made by the reasoner (excluding modeling mistakes)*; e.g., an unsatisfiable class resulting from disjoint ancestors that were aligned. While this may also have as source an ontological difference at the axiom-level, it would manifest either after adding the axioms, during test-driven development (TDD) [13], or upon ontology matching attempts.

What are the sources of such conflicts? The sources can be manifold and six principal cases were discerned, which are non-exclusive and possibly also not an exhaustive list:

- *Ontological differences between established theories*; e.g., extensional mereology vs. minimal mereology and DOLCE vs. BFO as top-level ontology.
- *Ontological differences at the axiom-level*; e.g., not all philosophers agree on whether the parthood relation is antisymmetric [19].
- *Different modeling styles*; e.g., foundational ontology-inspired or conceptual model-influenced, resulting in, e.g., reifying verbs into classes or adding them as object properties, respectively, [20], like the Vaccination/vaccinates mentioned in Fig. 1.

The first three are, in principle, *a priori* negotiations by an ontologist, but may manifest only upon ontology matching. Cases 4 and 5 emerge during ontology authoring. The last one may or may not be *a priori*. They will be elaborated on in the “Resolving conflicts” section further below.

#### The conflict set

Conflict detection offers opportunities for automation and, even though there is no single way of how conflicts can be detected, some tasks can be carried out with the aid of state-of-the-art ontology development environments (ODEs), which we elaborate on afterward and its requirements emanating from that in the “Discussion” section further below.

The data structure in which the detected conflicts are stored and upon which the resolution of conflicts operates, is called the *conflict set*, which is generated in all cases where a conflict is detected. Without loss of generality, it is assumed that, when matching more than two ontologies, a conflict set is generated for every pair of ontologies.

**Fig. 2:**
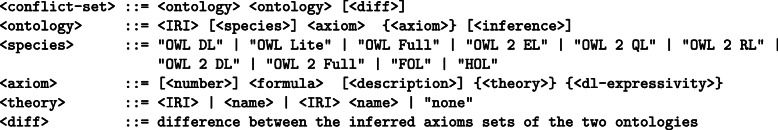
Conflict set grammar for recording individual conflict sets in or between ontologies (production rules of most terminals are omitted)

The conflict set is described in a context-free grammar in Backus-Naur Form, as shown in Fig. 2, for purposes of genericity, as such a grammar can be implemented easily in most programming and rule languages and enforce verification of correctness of any implementation of the conflict sets. Accordingly, there are two ontologies or two fragments of the same ontology, each identified by an IRI or another identifier and composed of a (possibly singleton) set of axioms. An axiom may adhere to an ontologically well-founded theory, such as ground mereology, or some domain theory. It is briefly illustrated in the following example with the Virus Infectious Diseases Ontology (VIDO) and the COVoc vocabulary, whereas a more comprehensive case is deferred to the case study in the “Results” section.

##### **Example 3**

The conflict set grammar of Fig. 2 is illustrated in ‘pretty printing’ notation with VIDO and COVoc, noting that we assume that $\mathsf {acellular\, structure} \sqcap \mathsf {organism} \sqsubseteq \bot $ is present in the ontology, following VIDO’s accompanying documentation [21] (though vido.owldoes not have that disjointness axiom), which is explained afterward:


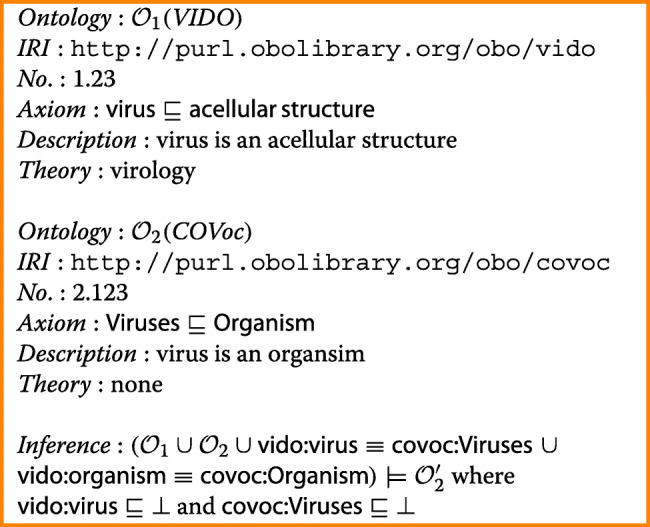


Both ontologies are identified with their respective < IRI >, for one conflict: in virology (the value of the conflict set’s < theory > for VIDO), viruses are acellular structures and not organisms, as shown with the relevant < axiom > number, axiom itself, and < description > thereof. The < inference > over the combined < ontology > (the “$\mathcal {O}_{1} \cup \mathcal {O}_{2}$” part in the box, above) with temporary name $\mathcal {O}_{2}^{\prime }$, and thus includes the two alignment axioms (the “ *vido*:*virus*≡*covoc*:*Viruses* and *vido*:*organism*≡*covoc*:*Organism*”), is that the respective virus classes are now unsatisfiable (${\mathsf {vido:virus}} \sqsubseteq \bot \ \text {and}\ {\mathsf {covoc:Viruses}} \sqsubseteq \bot $) and thus the combined ontology is incoherent.

The explanation thereof, which assists toward resolution of this conflict at the subject domain level, will be elaborated on in the next section, and its Fig. 5 in particular.

### Resolving conflicts

In practice, conflict resolution often starts with some issue raised by the ODE, and specifically when an axiom is added or an ontology is merged or integrated into the active ontology. Examples of such issues are *undecidability*, *language profile violation*, and *incoherence*. They can be seen as cues indicating that something is wrong with the active ontology. The author then has to find out what raised the issue. Thereby, they may be supported by the ODE. Proceeding that way is not as straightforward as one might expect, because there is no one-to-one correspondence between conflict and issue. Examples of such ‘causal investigations’ will be given in the following subsections. For the rest of this section, the following principal choices are presupposed:

- The ontology author adheres to Occam’s razor when developing an ontology for the case at hand: the least expressive language in which the required axioms can be represented fully is preferred over all more expressive ones.
- The ontology author wants to capture as much of the semantics of the domain theory as possible.
- The ontology author prefers a decidable language over first or higher order logic for representing a domain theory and a coherent ontology over an incoherent one (that suffers from at least one unsatisfiable class).

The first choice is a general principle in many situations in life. The second choice assumes that the author prefers representing a full axiomatization over a partial axiomatization and, by extension, a partial one is better than mere primitives without any axioms. While the third choice may not hold in all situations, we deemed it realistic to include, since most software infrastructure caters for decidable ontology languages and coherent ontologies, and Semantic Web and Knowledge Graph languages in particular.

We now proceed to discuss the conflicts listed in Table 1 in their order of presentation.

**Table 1:** A selection of conflicts that may emerge during ontology authoring, as a preliminary library of conflicts

No.	Conflict	Description	Examples
		*Conflicting theories at the top-level*	
1	foundational	ontologies adhere to conflicting theories	BFO, DOLCE, GFO, SUMO, UFO, YAMATO (see Table 2 for details)
2	mereological	conflicting mereological theories	with Atom or not, weak vs. strong supplementation
3	topological	conflicting topological theories	region connection calculus on non-simply connected regions
4	building blocks	different ontological commitments embedded in the language	whether roles are part of the fundamental furniture of the universe, 3D + time vs. 4D ‘worms’
		*Conflicting theories at the subject domain level*	
5	domain theory	theories with competing views of the whole domain	Newtonian physics vs. relativistic mechanics
6	status of an element	theories with competing views about a specific entity	whether virus is a living thing or not
		*Axiom-level conflicts*	
7	ontological	conflicting theories acting out on the axiom-level	pinpointing the violating axiom in items 1–3, 5, or 6, e.g., whether parthood is antisymmetric or not
8	within-language family	violation of a language profile beyond decidability	some of the non-admissible axiom combinations as listed in the first item of Example 4
		violation of a language profile, yet remaining decidable	functional and transitive properties in OWL 2 QL
		*Other conflicts*	
9	modeling style	applied vs. foundational	whether there are data property axioms, alike height between Person and xsd:decimal
		class vs. object property	Infection vs. infected-by
		subsuming roles vs. roles inhering in objects	doctor is-a person vs. doctor inheres-in person
10	language	cultural-linguistic and labeling differences, such as preferred/alt labels, orthography, language variants	population immunity vs herd immunity, eraser vs rubber, color vs colour, and non-1:1 mappings where a concept is named in one language but not in another (e.g., ‘river’ vs *fleuve* and *rivière*)

**Table 2:** Differences between foundational ontologies (non-exhaustive, partially based on ONSETv1.2 [7])

Feature	Examples
attributions	trope theory (UFO) vs. quality & qualia (DOLCE) vs. none (BFO)
stuff	yes (DOLCE) or no (BFO)
concepts	yes (DOLCE) or no (BFO)
abstract entities	yes (DOLCE, UFO) or no (BFO)
realizables	yes (BFO) or no (DOLCE, UFO)
functions	yes (BFO, YAMATO), no (DOLCE, UFO)
boundaries	yes (BFO) or no (DOLCE, UFO)
situations	yes (UFO, GFO) vs. no (BFO)
particulars & universals	both (UFO, GFO), either/or (DOLCE, BFO)

#### Conflicting top-level and domain theories

If an ontologically well-founded theory underlying some axioms to add or an ontology to integrate is in conflict with the ontology representing the desired theory (nos. 1–3 and 5 in Table 1), then the respective IRIs must be added to the conflict set. This presupposes that the conflict is known and the pair of IRIs is already listed somewhere, for instance, in a *library of common conflicts*. To give an example, if one wants to add the part-whole relations taxonomy [22] that is aligned with DOLCE to a BFO-aligned IDO [3], then the theory conflict ‘BFO vs. DOLCE’ will be detected through looking up the library of common conflicts. Conflict resolution, in this case, aims at preserving a consistent theory. Since for conflicting theories there is no joint outside option, the ontology author has to decide in favor of one theory and discard the other. Their decision may be informed by the deliberations of what should be represented in the ontology made during meaning negotiating.

ODEs support the import of ontologies and after import, their IRIs can be read from the metadata of the active ontology and looked up manually for common conflicts in a library. Accordingly, this conflict detection approach is straightforward to implement. Uncommon conflicts are harder to detect and therefore harder to resolve. The use of ontology design patterns [23] for a theory would be helpful in automating detection, as would annotations that describe which theory is being represented. In addition, the library of common conflicts may grow upon finding more conflicts, be this automatically or manually curated or both, so that it can prevent the same or a similar conflict from emerging later on in the project.

**Fig. 3:**
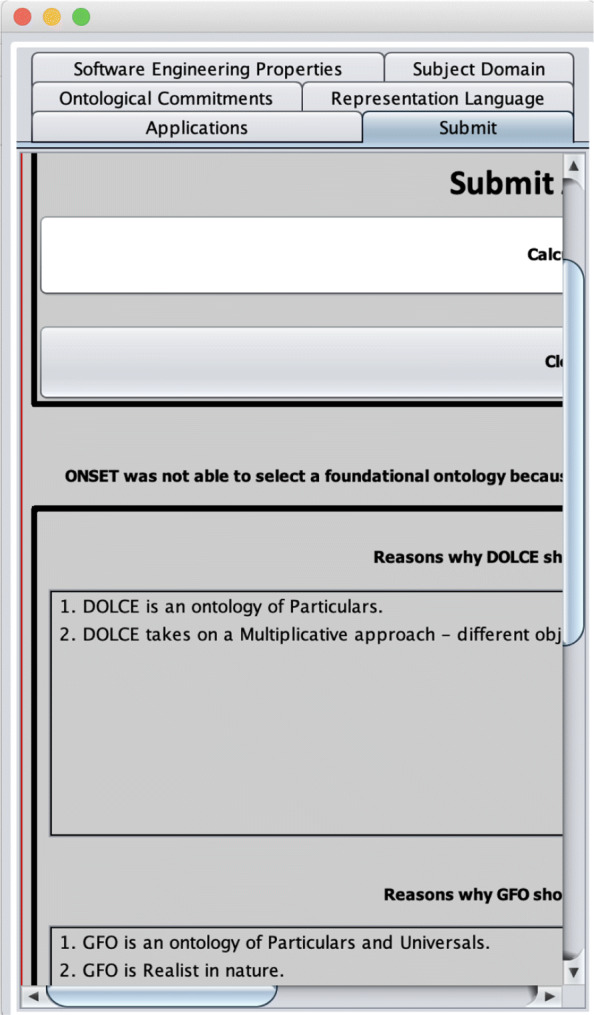
Explanation generated by ONSET [7] after having selected a combination of requirements for ontological commitments “Particulars, Realist, yet Multiplicative” that no foundational ontology can fully meet and whose conflict needs to be resolved at this requirements analysis stage

Taking a step back from practicalities like checking the IRIs, there may be ontological commitments at stake underlying differences in IRIs. A straightforward example of commitments and a foundational ontology that satisfies them would be “Universals, Realist, Concrete entities only”, which BFO meets [24]. One may have other preferences for commitments that do not agree fully. For instance, one may want an ontology for representing particulars, take a realist stance, yet also admit to a multiplicative approach where one admits to a difference between, e.g., the vase and the clay that it is made of. DOLCE and GFO, and SUMO each can only meet two of the three requirements, as illustrated in Fig. 3, and one then either has to change the requirements or accept that not all of them will be met and, in case of the latter option, which one is going to be traded in to compromise.

The top-level theory conflict listed in line No. 4 in Table 1 concerns ontologically irreconcilable differences that are embedded in the language that is used to represent the ontology in. This issue is described at length in [25] and its Table 2 lists the assessment of ontological features by ontology language, which can be applied for detecting conflicts. For instance, if one is convinced of the separation between natural language with labeling things one the one hand and the things themselves with their representation in logic on the other, then OBO is a good choice. Contrast this to OWL, which assumes the two are intertwined in that the classes and properties are expected to have human readable names. It is possible to convert one into the other, but it remains a brittle workaround due to the fundamental difference: practically, it acts out in ‘OBO style’ identifiers as names for classes and object properties in an OWL file with a hopeful wish that their labels are rendered in the tools. However, OWL assumes the elements are named and therefore labels are not mandatory, resulting in situations where the ‘OBO style forced into OWL’ occasionally breaks and one is left with only meaningless identifiers.

Conflicting theories at the subject domain level (nos. 5–6 in Table 1) may act out, and be detected and resolved, in two different ways. First, if each domain theory was represented in an ontology and had its own IRI, then one could use the same resolution approach as for the top-level theory issues of nos. 1–3 described above. Second, particularly in the case of fine-grained aspects like the status of an element, then they can be processed and resolved as for axiom-level conflicts, which will be described below and illustrated with a follow-up of Example 3.

#### Conflicts manifesting themselves in an undecidable language

With conflicts nos 7–8 in Table 1, conflict resolution aims at preserving a decidable ontology language or raising awareness of undecidability when opting for a joint outside option. In the first case, this most often is a zero-sum game: the ontology author has to chose which one(s) of the conflicting axioms in the conflict set to keep and which one(s) to delete. For mereotopological theories, these types of conflicts have been investigated [5]: in most instances, incorporating a full axiomatization renders the active ontology at least undecidable, and possibly also incoherent. To support the author’s decision, some criteria can be established for how to compute the possible resolution choices, such as the following:

- The least number of axioms are affected;
- The preferred axiom type is identified by assigning weights to classes, properties, or types of constraints;
- The least number of inferences are lost.

The *least number of axioms affected* can be read from the conflict set that was computed in detecting the conflict. Assigning weights to *axiom types* implies that certain types are *a priori* considered more valuable than others; e.g., one may weigh existentially quantified properties in axioms more than universally quantified properties and unqualified cardinality more than qualified cardinality. The *least number of inferences lost* requires an additional step where the inferences of the ontologies are computed and recorded in the conflict set. If undecidability is caused by an *ontological conflict* at the axiom-level that was not resolved along with conflicting theories (e.g., weak vs. strong supplementation in Example 4), then also the decisions taken when negotiating meaning upfront may serve as a criterion. The authors’ decision and the criteria upon which it is based ideally should be recorded so as to keep track of the decision made and in case the same or a similar conflict emerges later on in the project.

The second case, i.e., opting for a joint outside option, requires that principal choice (iii) (preferring a decidable language) is relaxed. Theories that are represented in different logics can be dealt with by the DOL framework [17]. This includes cases where the resulting logic is undecidable.

State-of-the-art ODEs provide some support for detecting and resolving these kinds of conflicts. To give an example, the OWL API [26] of Protégé 5 [27] issues an error message reporting the conflict arising from a violation of the expressive OWL 2 DL specification, which may render the language undecidable, when it is caused by a non-admissible axiom combination such as those listed in Example 4. In addition, Protégé 5 is equipped with an OWL reasoner, and a diff tool for computing the differences between OWL ontologies is available as a plug-in [28]. In order to compute the number of inferences lost, the axioms inferred from the merged ontology are first computed using the OWL reasoner. This requires that the merged ontology is saved as two decidable versions by removing one conflicting axiom set in exchange for the other. The difference between the sets of inferred axioms is then computed and returned to the modeler to inform them about the consequence of that particular choice.

##### **Example 4**

The following three examples are a sampling of mutually exclusive axiom combinations where a modeler thus has to choose either one or the other but they cannot be both in the same ontology:

- *Within language family:* an ontology expressed in OWL 2 DL where transitivity or a role chain is declared on some object property *R* prohibits the use of any of the following features to be declared on *R* as well, if the aim is to remain within OWL 2 DL: minimum cardinality, maximum cardinality, exact cardinality, functionality, inverse functionality, reflexivity, irreflexivity, asymmetry, and role disjointness.
- *Mereology:* weak supplementation, expressed as *pp*(*x*,*y*)→∃*z*(*p*(*z*,*y*)∧¬*o*(*z*,*x*)), in the mereological theory called Minimal Mereology is incompatible with strong supplementation (¬*p*(*y*,*x*)→∃*z*(*p*(*z*,*y*)∧¬*o*(*z*,*x*))) in Extensional Mereology.
- *Temporal logics:* one has to choose how to ‘see’ time, where it is either discrete such that there is a first and last time point *t* and a series of successive time points with no time point between any *t* and *t*+1 or it is dense such that ∀*t*,*t*^′^∈*T*,*t*<*t*^′^,∃*t*^″^.*t*<*t*^″^<*t*^′^, i.e., it is infinitely possible to squeeze another time point between two adjacent time points.

#### Conflicts manifesting themselves in a language profile violation

This may occur in particular with conflict no 8 of Table 1, but may also appear as a confounding issue in conflict 7. The case where resolution aims at preserving the language to the extent that it is decidable again, while accepting that the original profile is violated, has to be distinguished from the case where the original language profile should be preserved. Presupposing aforementioned principal choices, conflict resolution in the first case aims at capturing as much of the semantics of the domain theory as possible. Since the language profile that was violated may not be the most expressive one, there may be room for a (decidable) joint outside option. To give an example, the ‘overlap’ axiom *O*(*x*,*y*)=_*def*_∃*z*(*P*(*z*,*x*)∧*P*(*z*,*y*)) cannot be expressed in any decidable OWL species, because it does not admit property definitions in the language. That is, the syntax does not permit usage of equivalence for object properties and, in fact, even puts limitations on property subsumption so as to prevent cycles in the hierarchy (called a “regular” role hierarchy [29]). While preserving decidability, the author may still want to state that *P*(*z*,*x*)∧*P*(*z*,*y*) is a sufficient condition for *O*(*x*,*y*) or they may want to state that *O*(*x*,*y*) is a reflexive and symmetric property. Yet, doing so may violate the original language profile. Whether the ontology language still is undecidable with the modified axioms and conditions can be figured out in the same way as described in the previous section, about Conflicts Manifesting Themselves in an Undecidable Language. Weakening the theory step by step this way will end up in a representation such that the language features used remain within a decidable language, since the representation of properties as mere primitives is always possible in OWL and other ontology languages.

In the second case, conflict resolution aims at preserving the original language profile at the expense of relaxing principal choice (ii), i.e., accepting that ‘as much semantics as possible’ is less than anticipated. This applies to conflicts emerging from what is called *design for scalability* in the “Types and sources of conflicts” section, above. Here, conflict resolution is likely to be a zero-sum game similar to that described in the section about undecidability. For instance, the OWL 2 QL profile is aimed at applications that use very large amounts of data, such as conventional relational database systems, and where query answering is the most important reasoning task. Violating this profile then means accepting that query answering may no longer be implementable by rewriting queries into a standard relational query language [30].

Concerning tool support for these two cases, in the first case, it is the same as that described in the preceding section about undecidability. For the second case, the OWL Species Classifier supports authors of OWL ontologies by listing which axioms violate which OWL species (see footnote 1). An illustration of the benefits of this sort of automation is described in the following example, where the classifier was used to search through the 417 axioms of the CIDO ontology for COVID-19 [31] to check for profile violations, compared to manually searching for a possible needle in a haystack.

##### **Example 5**

**Fig. 4:**
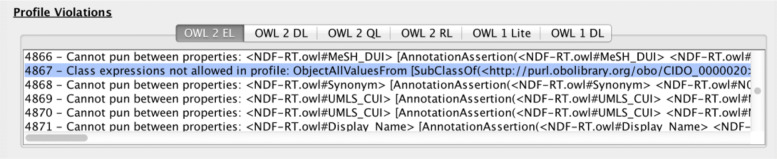
Section of the OWL classifier, having detected that CIDO_0000020, i.e., Yale New Haven Hospital SARS-CoV-2 assay, is one of the class expressions violating the OWL 2 EL profile

Practically, medical ontologies for information systems typically do not exceed the OWL 2 EL profile, because of scalability and compatibility with typical OBO Foundry ontologies and SNOMED CT, so let us assume this as a requirement. CIDO [31] is not within OWL 2 EL, however, which is due to a class expression with a universal quantifier on the right-hand side; more specifically, as easily pinpointed by the OWL Species Classifier (see Fig. 4), ‘Yale New Haven Hospital SARS-CoV-2 assay’ $\sqsubseteq \forall $’EUA-authorized use at’.’FDA EUA-authorized organization’ is one of the axioms that violate the OWL 2 EL expressiveness restrictions not only in the initial unofficial cido.owl of 14 June 2020 but also still in the most recent release of 31-1-2021 (v1.0.181); see also Fig. 4.

#### Conflicts manifesting themselves in an incoherent ontology

**Fig. 5:**
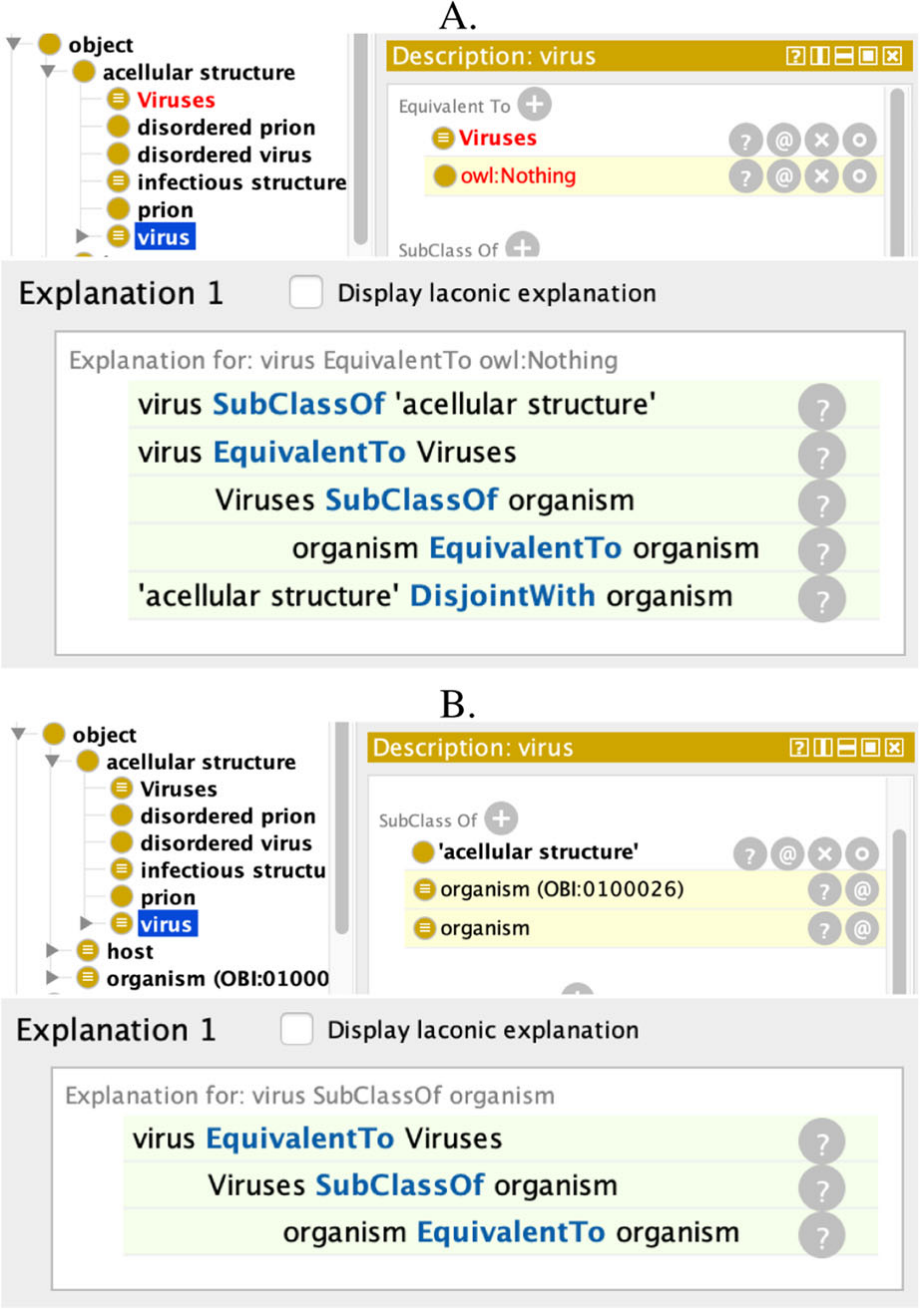
Inference visualisation and justification algorithm explanations of [11] as implemented in Protégé v5.5. A: explanation in the case of the conflict described in Example 3; B: explanation of inference with the VIDO and COVoc ontologies as-is with the two alignment axioms on virus and organism

Conflicts nos. 7–8 in Table 1 may also manifest themselves as an incoherent ontology, with one or more unsatisfiable classes or properties (observe that conflict no. 7 includes any remaining unresolved theory-level conflicts of nos. 1–3, 5, and 6). Conflict resolution in this case aims at preserving or obtaining a coherent ontology. Examples include ontological misspecifications at the axiom-level, such as disjoint ancestors, resulting in unsatisfiable classes (no. 7 in Table 1). Such conflicts manifest only when the automated reasoner reports the deductions; an example is shown in Fig. 5. In the simplest case, they are resolved by keeping some of the conflicting axioms and removing others in a way similar to that described in the section about undecidability, above. Typically, either the disjointness axiom on the ancestors or the subclass axioms on the class may be kept, but not both. State-of-the-art ODEs allow for making deductions. After running the OWL reasoner in Protégé 5.x, for instance, unsatisfiable classes and properties are displayed in red color. In order to find out what made them unsatisfiable, justifications can be computed using the respective plug-in, where a *justification* is a set of axioms from an ontology that is sufficient for an entailment to hold [11]. In the case of unsatisfiable classes and properties, justifications are computed for entailments with owl:Nothing and owl:bottomObjectProperty on the right-hand side of the inclusion axiom, enabling identification of the source(s) of incoherence.

#### Conflicting modeling styles and language issues

These conflicts arise from source 3 listed at the start of the “Methods” section and concern the ‘other conflicts’, being nos. 9–10 listed in Table 1. Resolving them aims at restructuring (parts of) an ontology such that correspondences with entities of a different ontology can be established. For instance, if the same notion is modeled in one ontology as a class and in another as an object property (see Fig. 1), or even in both ways in the same ontology, and the ontology language does not permit heterogenous alignments, then either the object property has to be reified or the class has to be recast as an object property (see no. 9 in Table 1). Typical examples are object properties such as o1:married-to and o1:has-member and corresponding reifications as o2:Marriage and o2:Member, respectively. A concrete difference in modeling choice in two ontologies in the same domain is illustrated in Example 6.

##### **Example 6**

Let us continue with COVID-19 ontologies. Besides the CIDO (v1.0.181), there is also the CODO [32] (v1.3 of 25-9-2020), which focuses predominantly on patient data and therewith alike a practice-oriented application ontology. CODO’s COVID-19 test results are represented as codo:‘laboratory test finding’ ≡ {positive, pending, negative}, i.e., the outcomes are instances and, within the context of the other content in the ontology, practically conflated with the diagnosis of the disease. In contrast, CIDO is informed by the BFO top-level ontology with its modeling guidelines and has a cido:‘COVID-19 diagnosis’ class with three subclasses: negative COVID-19 diagnosis conclusion, and one for positive and one for presumptive positive. Aside from disagreeing on names and possibly also the meaning of possible test statuses, the modeling style issue here is that it is an example of class vs. instance modeling conflict of the same notion. Ontologically, the same notion cannot be both a class and an instance and should thus not co-exist in one ontology, hence, one of the two options will have to be chosen if they were to be integrated.

What is recorded in the conflict set depends on the case at hand; for the class vs. property example, these would be the respective axioms to match and the axioms they are used in, which may be found by using a natural language processing-based algorithm with part-of-speech tagging and stemming. Generally speaking, there are two different options of dealing with conflicting modeling styles. The first is to choose one style and then convert instances of the ‘losing’ modeling pattern into the style of the ‘winning’ pattern, where each consists of one or more axioms, and the second option is to keep both styles and match patterns by a set of axioms, rather than by a single bridging axiom, which is referred to as a heterogeneous TBox mapping [20].

The way how ODEs deal with conflicting styles depends on the kind of conflict and the ODE. For instance, Protégé 5 restricts alignments to equivalence and simple subsumption between classes and between object properties. Simply put, it does not provide the necessary means to assert an alignment between a class and an object property. An algorithm for detecting conflicting modeling styles and additional axioms for heterogeneous alignment has been proposed [20], but this is yet to be integrated in an ODE. A joint outside option may be the DOL framework that already does have a logic-based mechanism for heterogeneous alignments [17].

Language conflicts (no. 10 in Table 1) may vary in severity. There are straight-forward differences in naming patterns, such as camel case naming versus using underscores between words in a term, which are detected by the OOPS! tool [33], and one can use a spelling checker to detect US English versus British English orthography and more advanced resources to assist with synonym detection and resolution. It will not yet resolve which English variant to use in an ontology. More detailed labeling of language aspects and a stricter separation of the language layer on top of the ontology layer may assist in resolving this. Promising proposals for such additional modeling are the Ontolex-lemon community standard [34] and the MoLA [35] language annotation model. Then, instead of an either-or choice for one way of labeling, it can be both-and with an arbitrary number of alternative labels.

## Results

The framework proposed here is tested against a realistic case of epizootic disease outbreak in the Lemanic Arc (France, Switzerland) in 2006 [36]. To this end, case records of three occurrences of human-pathogenic avian influenza (H5N1) in wild birds were examined. The measures taken by the Swiss authorities to prevent the virus from infecting domestic poultry consisted of establishing protection zones within a radius of at least 3 kilometers and surveillance zones within a radius of at least 10 kilometers. In these zones regulations, such as poultry must be kept in the henhouse, were introduced. The Swiss authorities had to decide which municipalities to include in the protection zones and which in the surveillance zones.

**Fig. 6:**
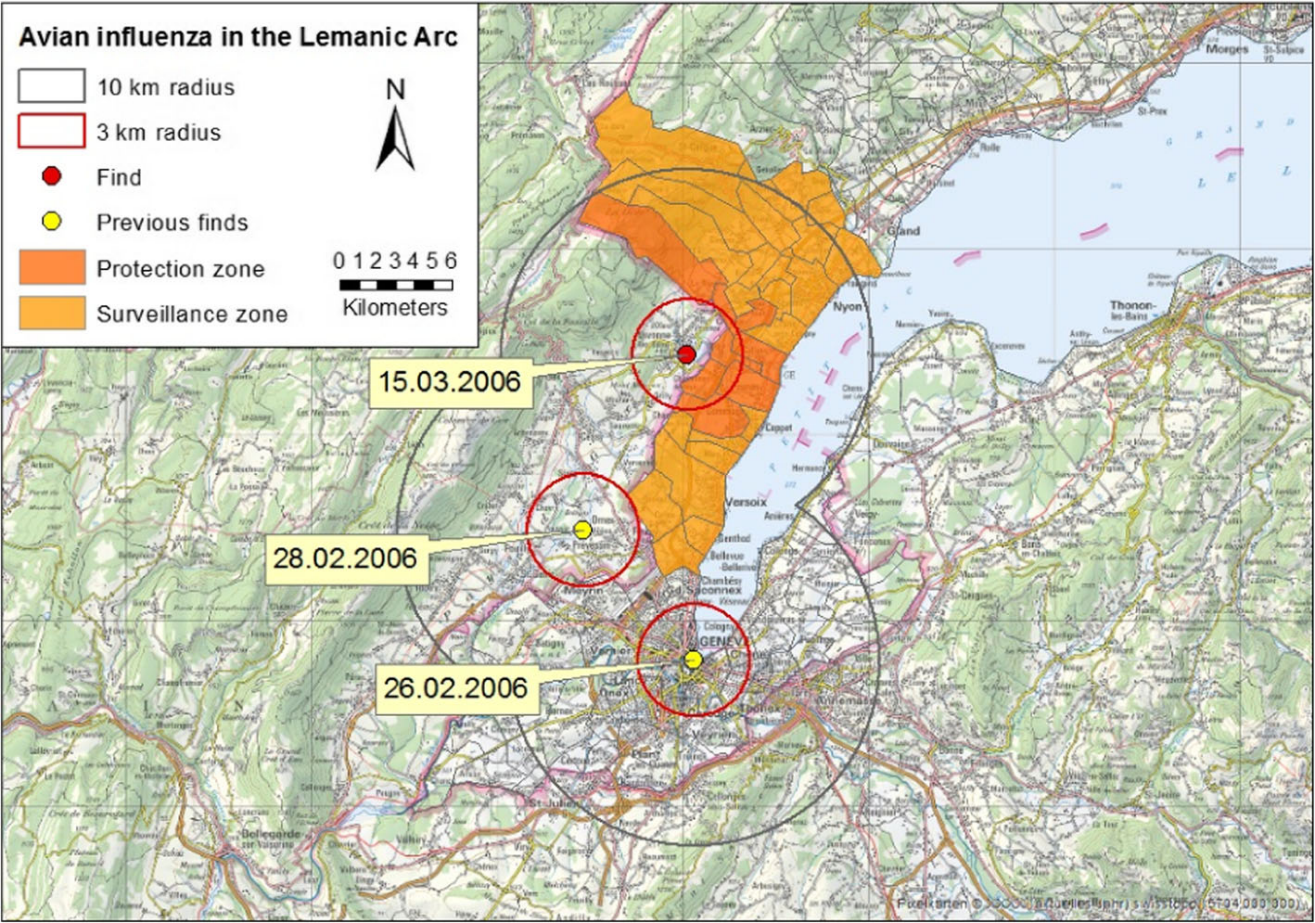
Avian influenza in the Lemanic Arc (adapted from [37]). National Map 1:200,000 Ⓒ 2008 swisstopo

Figure 6 shows the second last stage of the avian influenza outbreak. Since the regulations brought into operation after the first two occurrences are no longer in effect, the affected municipalities are not highlighted, but those in the protection and surveillance zones of Divonne-les-Bains (Ain, France).

Assume the administrative division of Switzerland is represented in administrative ontology $\mathcal {O}_{1}$ and the finds of infected birds as well as protection and surveillance zones are represented in epidemiology ontology $\mathcal {O}_{2}$ (only the RBoxes are displayed; the ontologies can be downloaded from https://www.envidat.ch/dataset/icbo2020). In order to construct a query against a geodatabase to figure out which municipalities to include in which zones, the two ontologies need to be merged. Both are OWL 2 DL ontologies with an expressivity of $\mathcal {ALCRIF}$ and $\mathcal {SRIF}$, respectively. They have been implemented using Protégé 5.2 [27]. *District*,*Municipality*,*Find*,*Zone* (not shown) describe classes of objects that are related to each other by roles *located*_*in*,*located*_*in*_*inv*,*proximal*. Their spatial extensions are instances of the class *Region* (not shown) which are interrelated by roles *partOf*,*overlaps*. Roles *has*_2D,*has*_2D_*inv* relate objects to their spatial extensions and vice versa.

In order to represent the administrative division properly, every region occupied by a municipality is assigned to exactly one region occupied by a district (line 1.22). Accordingly, the role *partOf* is functional in ontology $\mathcal {O}_{1}$. For the finds of infected birds in ontology $\mathcal {O}_{2}$, on the other hand, the same role needs to be transitive (line 2.32): The (small) regions occupied by the finds are contained in the regions occupied by the protection zones. These are contained in the regions occupied by the surveillance zones. Merging the two ontologies, thus, results in a conflict which is reported by the following conflict set:


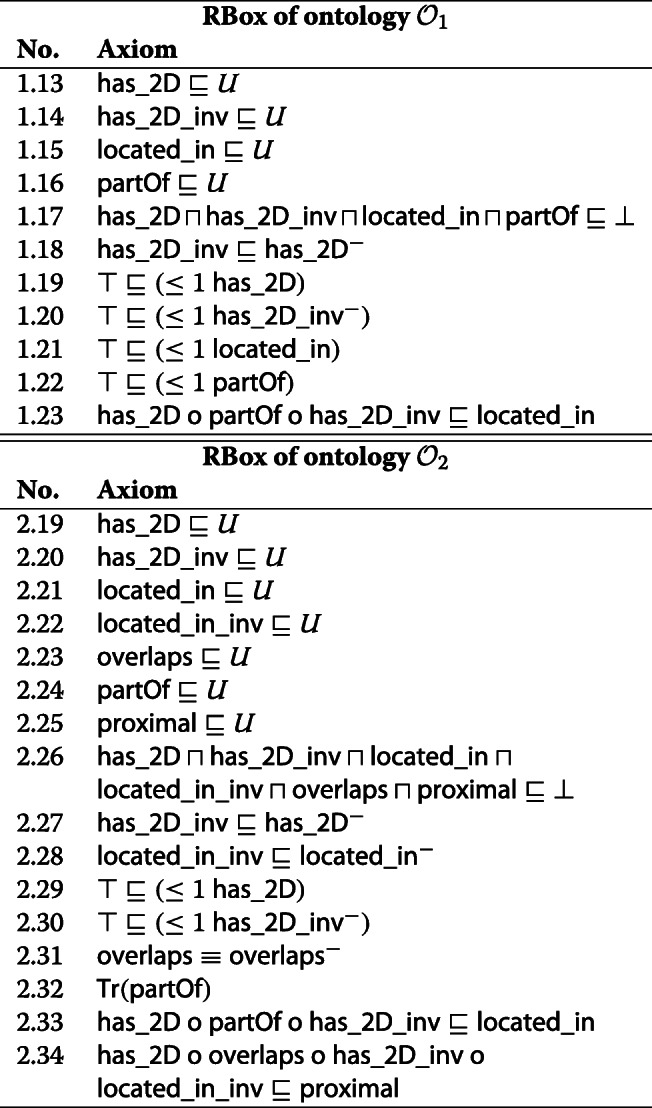



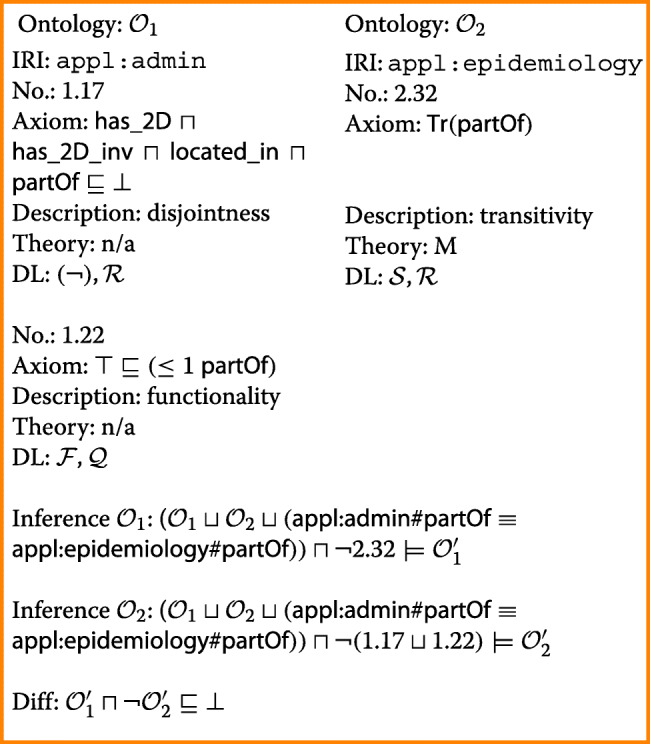


The conflict is resolved by trading transitivity of appl:epidemiology#partOf for functionality of appl:admin#partOf and disjointness of roles in the administrative ontology. Doing so affects less, but equally preferred, axioms than the other way round, namely, one axiom vs. seven axioms (please note: ‘axiom’ 1.17 is a shorthand notation for six individual axioms omitted due to space limitations). It loses exactly the same inferences (diff is empty) as trading in the opposite direction.

## Discussion

**Fig. 7:**
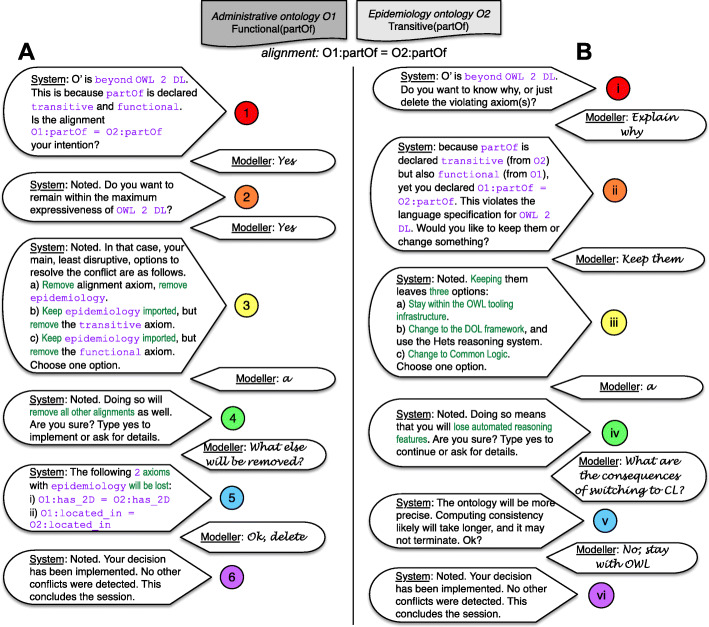
Two types of cognitive walk-throughs for the Avian influenza Case Study. A: flat iteration from detection (1) to presenting options (2 and 3), interactions about the consequences of the choice made (4), and to implement the decision (5). B: begin with core choice (i), where an explanation can be given for those who want it (ii), and likewise with basic options (iii) and details if explanations are wanted (iv and v), and closing with implementing the choice (vi). The text in Courier font (purple) is content fetched from the conflict set data structure and the snippets in Arial Narrow (green) could be values of variables fetched from structured information about typical resolution options

A first step for a conflict resolution framework has been made, comprising the basic specification what it is, a specification of a conflict set, a first library of conflicts, and indications of paths toward resolution. Examples and the case study suggest that conflicts are abound already, and this is likely set to increase with the increasing number of ontologies that are being developed. This, in turn, brings afore an imperative for software-supported conflict detection and resolution to further systematize and facilitate the process. Broadly, for each type of conflicts one needs (1) mechanisms—software tools, where possible—to be able to detect them; (2) to store the information collected upon detection for further processing; (3) a set of resolution strategies associated with each type of conflict that then can be used in some form by the user, for instance, in a Query & Answer dialogue, alike demonstrated in Fig. 7; and (4) a means to automate the implementation of the choice made. We discuss the requirements for such software support in the remainder of this section, first specifically for the walk-throughs of the case study, and then more generally.

### Tool requirements for the walk-throughs

A system encompassing the whole walk-through of the case study, alike in Fig. 7, requires, as a minimum, a Question Answering system that avails of a controlled natural language or a natural language generation system to generate the text and to process the user’s input.

For step 1/i, the “beyond OWL 2 DL” is a slot filled with that value in a template sentence “O’ is [*species*].”, where the value for the *species* variable is fetched from the conflict set, and likewise for the “partOf” etc that are values from the < axiom > variables of the grammar (recall Fig. 2). This entails the software requirement to be able to find such conflicts, which, in this case, can be achieved with the OWL Species Classifier and processing of its output into the conflict set data structure, and additional template design for a ‘pretty printing’ wrapper. This is likewise for generating the explanation in step ii.

Steps 2 & 3 and ii & iii require a mechanism to propose alternatives, which has to consist of at least two components: a data structure storing the typical resolutions that may be feasible for each conflict and a decision tree covering each option. For instance, there are 6 possible choices for the case study (yes + a, yes + b, etc.): for the “yes” choice in walk-through A, the options are always to roll-back the integration, delete one or delete the other axiom, whereas the “keep” answer in step ii, i.e., to keep the axioms (in walk-through B), has three choices that can be presented as canned text.

Once at the end of the decision tree, step 4/iv and 5 require an algorithm to gather all consequences of each leaf in the decision tree. Consequences may be only a list of axioms deleted or also inferences lost, which have to be collected on-the-fly. Such algorithms will have to be developed. For step 4’s roll-back, it simply means using the OWL API [26] or Owlready [38] to query the ontology for alignment axioms between the two ontologies (that are identifiable with their different IRIs) and displaying the query answer by slotting in those axioms into a variable length template. If the resolution choice would also show the deductions lost or options b) or c), then it will take some more effort, in that the ontology then has to be copied, the relevant axiom removed, inferences computed and the differences in entailments determined (e.g., with the OWL Diff tool [28]), which then would be rendered. No tool exists that does this whole process, but the key components exist. Canned explanations, as in step v, obviously can be prepared easily.

The last step, 6/vi, sounds deceptively simple, but the ease or difficulty to implement it depends on the type of conflict and the choice. For option a) in walk-through A, as well as for the earlier example about viruses in Fig. 5: if one has chosen to remove the ontology or the offending axiom, then it is a simple deletion of the axioms identified for step 5. Ignoring the reasoner (step vi) is even easier to do.

### Further tool requirements

The implementation of the choices in Fig. 7 are straight-forward. It rapidly can become more challenging for other chosen resolutions, however. For instance, a modeller may have chosen the DOLCE ontology from the choices shown in Fig. 3, then the implementation of that choice depends on the starting position: perhaps it is simply an import of DOLCE-lite.owl, but if another foundational ontology was used before, it would entail swapping out the old foundational ontology for DOLCE and aligning one’s domain entities to that, either manually or automatically with SUGOI [39]. The remodeling of all vaccinates object property usages in axioms into its reified Vaccination, as suggested in Fig. 1, requires a substantial modification of the ontology between two modeling patterns for which no algorithm exists yet.

Alternative routes may be possible for other conflicts and walk-throughs, such as assisting with testing the effect of axioms oneself with TDDonto2 [13], and migration paths to the ‘beyond OWL’ infrastructures, such as OntoHub [40] with the Hets toolset reasoner for ontologies in the DOL framework [41]. The requirements for advanced handing of language conflicts require software support for, e.g., the Ontolex-lemon [34] and MoLA [35] models, which presuppose that such issues can be detected, which is also a requirement to be fulfilled. Similarly, there are theoretical advances on modeling styles [20] for detecting and resolving conflict 9 of Table 1 and for ontological conflicts between modeling languages [25] to resolve conflict 4 of Table 1: also here the main requirement is to repurpose the theory for software-supported conflict resolution.

To sum up, the minimal system requirements are:

- A conflict resolution workflow management system, be it a Question Answering system or another strategy that avails of a knowledge-to-text controlled language, canned text, a decision tree, and two data structures (the conflict set and the resolution options);
- Algorithms to populate the conflict set, which may avail of new wrappers for existing OWL tools to recast their computation and outputs as detection and conflict resolution functionalities;
- End-user usable DOL and CL tools;
- Software support for the language annotation models and extant assessments on modeling style and language conflicts.

From a different viewpoint: these requirement are future work. The first steps on the conflict resolution path that we presented here, together with the case study, has enabled a formulation of tool requirements, paving a path forward.

## Conclusions

Foundational steps towards a framework that can deal in a systematic way with the various types of modeling conflicts through meaning negotiation and conflict resolution have been proposed. The article introduced and specified the notions of meaning negotiation and conflict resolution, outlined what their components are, and presented a first step towards a library of conflicts. There is no single way of detecting and resolving conflicts, where four common categories were described, with conflicts at the top-level theory level, at the subject domain level, and axiom-level conflicts, which intersect with language expressiveness conflicts. The notion of the conflict set was also introduced, which is a minimal data structure in which the detected conflicts can be stored and upon which a software-mediated conflict resolution will be able to operate. This approach was evaluated with an actual case of domain knowledge usage in the context of epizootic disease outbreak. The theory and use case combined assisted in elucidating software requirements for conflict resolution workflows.

While there are some tools and plugins that can assist with meaning negotiation and conflict resolution, no integrated support is currently provided. Future work includes refining the framework and proceed to components of automating conflict detection and resolution, as well as establishing the conflict library in more concrete terms.

## Data Availability

The ontologies of the case study are available at https://www.envidat.ch/dataset/icbo2020.

## Abbreviations

API: application programming interface
BFO: basic formal ontology
CIDO: coronavirus infectious disease ontology
CLIF: common logic interchange format
CODO: covid-19 ontology
DOL: distributed ontology mode and specification language
DOLCE: descriptive ontology for linguistic and cognitive engineering
FOL: first order (predicate) logic
GFO: general formal ontology
HOL: higher order logic
IDO: Infectious Diseases Ontology
IRI: internationalized resource identifier
ODE: ontology development environment
OWL: web ontology language
SNOMED CT: systematized nomenclature of medicine – Clinical Terms
SUMO: standard upper merged ontology.

## Footnotes

1 https://github.com/muhummadPatel/OWL_Classifier/
